# Quantification of the effect of instrumentation error in objective gait assessment in the horse on hindlimb symmetry parameters

**DOI:** 10.1111/evj.12766

**Published:** 2017-11-01

**Authors:** F. M. Serra Bragança, M. Rhodin, T. Wiestner, E. Hernlund, T. Pfau, P.R. van Weeren, M. A. Weishaupt

**Affiliations:** ^1^ Department of Equine Sciences Faculty of Veterinary Medicine Utrecht University Utrecht The Netherlands; ^2^ Department of Clinical Sciences Swedish University of Agricultural Sciences Uppsala Sweden; ^3^ Equine Department Vetsuisse Faculty University of Zurich Zurich Switzerland; ^4^ Department of Clinical Science and Services The Royal Veterinary College Hatfield Hertfordshire UK

**Keywords:** horse, gait analysis, lameness, symmetry, objective, marker placement

## Abstract

**Background:**

Objective gait analysis is becoming more popular as a tool assisting veterinarians during the clinical lameness exam. At present, there is only limited information on the effect of misplacement of markers/motion‐sensors.

**Objectives:**

To investigate and describe the effect of marker misplacement on commonly calculated pelvic symmetry parameters.

**Study design:**

Experimental study.

**Methods:**

Each horse was equipped with custom‐made devices consisting of several reflective markers arranged in a predefined manner with a reference marker correctly positioned regarding the anatomical landmark and several misplaced markers along the sagittal and transverse planes. Linear regression analysis was used to estimate the effect of marker misplacement.

**Results:**

For the tubera sacrale, each cm of left/right misplacement led to a difference in minimum position of the pelvis (PDmin) of ±1.67 mm (95% CI 1.54–1.8 mm) (P<0.001); maximum position of the pelvis (PDmax) was affected by ±0.2 mm (95% CI 0.071–0.33 mm) (P = 0.003). With respect to cranial/caudal misplacement, each cm of misplacement resulted in a PDmin difference of ±0.04 mm (95% CI −0.09 to 0.16 mm) (P = 0.56) and a PDmax difference of ±0.008 mm (95% CI −0.13 to 0.12 mm) (P = 0.9). For the tubera coxae, each cm of vertical misplacement led to a difference in the displacement amplitude between left and right tubera coxae (Hip‐Hike_Diff) of ±1.56 mm (95% CI 1.35–1.77 mm) (P<0.001); for the cranial/caudal misplacement, this was ±0.82 mm (95% CI 0.66–0.97 mm) (P<0.001).

**Main limitations:**

Only three horses were used in this experiment and the study design did not permit to determine the influence of marker misplacement on the evaluation of different degrees of lameness.

**Conclusions:**

Marker misplacement significantly affects calculated symmetry parameters of the pelvis. The observed errors are overall small but significant. In cases of mildly asymmetrical horses, this error might influence the decision‐making process whereas in more severe asymmetries, the influence of the error effect may become less significant.

## Introduction

Kinematic gait analysis has the potential to provide clinicians with accurate and unbiased information that can be used during orthopaedic examinations of horses. This technique is also routinely used in equine research as a method of objective quantification of locomotion and to quantify gait changes due to orthopaedic pain. Its clinical application helps overcoming the inherent limitations of subjective lameness assessment, mainly the low inter‐observer agreement 1, 2, 3, 4 and human limitations of visual asymmetry perception 5, 6. In recent years, several clinically applicable methods have become available and these are in general based on the evaluation of asymmetries of the vertical displacement of head, withers and pelvis 7, 8, 9, 10, 11, 12 during unridden trot.

Kinematic gait analysis relies on the placement of sensors or markers attached to the skin over predetermined anatomical landmarks. Although the repeatability of most symmetry parameters has been evaluated to a certain extent 7, there is only limited information 13, 14 about how misplacement of markers might affect the measured symmetry parameters and ultimately influence the decision‐making process. Previous research demonstrated that marker placement is crucial when assessing locomotion asymmetries using limb mounted markers 15 and that small differences in marker placement can indeed create artificial asymmetries in the measured outcome. Skin displacement artefacts due to the displacement of the skin are also a known issue in equine gait analysis and can have a major effect on kinematic measurements of the limbs 16, 17 and to a certain extent also of the tubera coxae and sacrale 18.

The objectives of this study were to investigate to what extent misplacement of markers for kinematic gait analysis might influence the commonly calculated symmetry parameters used for hindlimb lameness quantification. Our hypotheses were that: 1) marker misplacement will have a significant effect on all measured symmetry parameters and 2) such effect will be more important when the misplacement is out of the sagittal plane.

## Materials and methods

Animals: Three riding school horses (Horse ID: 1, 2 and 3) were used in this study (one German Warmblood, one German Riding pony and one Fjord horse) with a height at withers of 1.70 m, 1.48 m and 1.44 m, respectively, a body mass of 640 kg, 410 kg and 520 kg, respectively and ages of 7, 11 and 15 years, respectively. Horses had been accustomed beforehand to treadmill locomotion as previously described 19. Horses were in regular use and known to be mildly asymmetrical. Measurements for objective lameness assessment can be found in Table 1.

**Table 1:** Baseline symmetry parameters calculated for each of the study subjects for head and pelvis. HDmin/HDmax: Head minimum/maximum position difference, PDmin/PDmax: Pelvis minimum/maximum position difference, HD_SI__up_/HD_SI__down_: Head upwards/downwards vertical displacement difference index. PD_SI__up_/PD_SI__down_: Pelvis upwards/downwards vertical displacement difference index. Values are mean and ±standard deviation in brackets

Horse	Warmblood horse	Riding pony	Fjord horse
HDmin	−6.1 (25.7) mm	−16.5 (19.5) mm	1.6 (10.9) mm
HDmax	12.7 (15.4) mm	11.5 (16.1) mm	9.9 (7.9) mm
HD_SI__up_	0.06 (0.37)	−0.1 (0.5)	0.1 (0.2)
HD_SI__down_	−0.3 (0.4)	−0.5 (0.3)	−0.1 (0.1)
PDmin	13.3 (6.0) mm	−5.2 (5.7) mm	−6.2 (5.4) mm
PDmax	−0.1 (3.9) mm	4.1 (7.2) mm	−8.2 (5.7) mm
PD_SI__up_	0.2 (0.1)	−0.01 (0.1)	−0.2 (0.1)
PD_SI__down_	0.2 (0.1)	−0.1 (0.1)	0.03 (0.1)
Hip‐Hike Diff	21.4 (8) mm	0.6 (9.5) mm	−11.5 (10.9) mm

**Figure 1:**
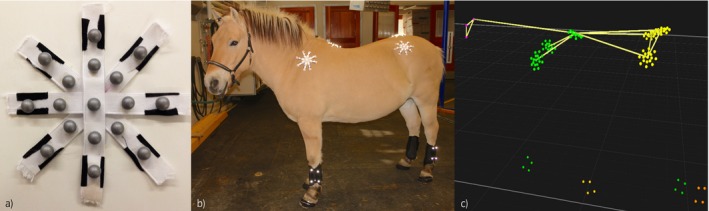
Marker experimental setup. a): Reflective marker cluster construction. b) Marker placement in one of the study subjects. c) Example of left lateral view of all 121 markers as depicted by the 3D tracking software.

Marker placement: 121 spherical reflective markers were used, with a cluster of three markers placed on the head (20 mm∅), a cluster of four markers (15 mm∅) on the mid‐lateral aspect of each metacarpal/metatarsal bone aligned with the bone longitudinal axis, and star clusters of 17 markers (12 mm∅) each on the left tubera coxae (LTC), right tubera coxae (RTC), sacrum, withers and left and right tubera of the spina scapulae, respectively (Fig 1). For this investigation, only the sacrum, LTC and RTC cluster markers were analysed. The central marker of each cluster was positioned over the correct anatomical landmark (sacrum, centrally between the cranial aspects of the tubera sacrale; LTC/RTC, dorso‐cranial aspect of the tubera coxae) and one of the arms of the star‐shaped figure was aligned with the transverse plane of the horse at that position. The label name of each marker can be found in Table 2.

**Table 2:** Naming of the different markers for each cluster location in relation to the horse, relative to global‐coordinate system (treadmill)

Location/Naming	Sacrum	LTC/RTC
0	Reference marker	Reference marker
a/−a	±3 cm displacement. Left (+), Right (−)	±3 cm displacement. Proximal (+), Distal (−)
b/−b	±6 cm displacement. Left (+), Right (−)	±6 cm displacement. Proximal (+), Distal (−)
1/−1	±3 cm displacement. Cranial (+), Caudal (−)	±3 cm displacement. Cranial (+), Caudal (−)
2/−2	±6 cm displacement. Cranial (+), Caudal (−)	±6 cm displacement. Cranial (+), Caudal (−)
I/−I	±3 cm displacement. Left/Caudal (+), Right/Cranial (−)	±3 cm displacement. Cranial/Distal (+), Caudal/Proximal (−)
II/−II	±6 cm displacement. Left/Caudal (+), Right/Cranial (−)	±6 cm displacement. Cranial/Distal (+), Caudal/Proximal (−)
α/−α	±3 cm displacement. Left/Cranial (+), Right/Caudal (−)	±3 cm displacement. Cranial/Vertical (+), Caudal/Distal (−)
β/−β	±6 cm displacement. Left/Cranial (+), Right/Caudal (−)	±3 cm displacement. Cranial/Vertical (+), Caudal/Distal (−)

### Data collection

Kinematic data were collected at trot at 3.6 m/s (treadmill belt speed) using 10 infra‐red 3D motion capture (MoCap) cameras (Oqus 700+/300+ and 400)^1^. Force data were recorded with an instrumented treadmill^2^, ^20^. Sampling frequency was 400 Hz for force and 200 Hz for MoCap data. Both measurement systems were synchronised using hardware synchronisation. Each recording lasted 20 s.

### Data processing

During the measurements, the three‐dimensional coordinates of each marker were automatically tracked by the motion capture software (QTM, version 2.13)^1^. After each measurement, visual inspection of the 3D tracked data confirmed that all markers were properly tracked and data were suitable for analysis. Measurements with poor marker tracking or irregular gait patterns were discarded and repeated. All data were exported into a Matlab^3^ file. Custom‐made Matlab scripts^3^ were used to process all data and calculate symmetry parameters for each marker of each cluster. Stride split was performed using hoof contact timings based on the kinetic data. The vertical displacement of each marker was high‐pass filtered using a 4th order zero‐phase Butterworth filter with the cut‐off frequency (frequencies used: 1.1, 1.0, 0.95 Hz) adjusted to the stride frequency of each trial/horse (stride frequency: 1.6, 1.5, 1.4 Hz, respectively). For each marker, the calculated symmetry parameters were the pelvis PDmin/PDmax, PD_SI_up_/PD_SI_down_ and Hip‐Hike difference as described in Table 3. All parameters were calculated for each stride as previously described 21.

**Table 3:** Detailed description of the calculated asymmetry parameters used in this study

Parameter	Description
PDmin	Difference between left and right stride half‐cycle in minimum vertical displacement/position of the sacrum in mm.^a^
PDmax	Difference between left and right stride half‐cycle in maximum vertical displacement/position of the sacrum in mm.^a^
PD_SI_up_	Symmetry index of the upward pelvis vertical displacement as a proportion of the absolute pelvis vertical range of motion of each stride.^b^
PD_SI_down_	Symmetry index of the downwards pelvis vertical displacement as a proportion of the absolute pelvis vertical range of motion of each stride.^b^
Hip‐Hike difference	The difference in the upwards amplitude of the vertical displacement between the left tubera coxae and the right tubera coxae in mm.^a^

a A value of 0 indicates perfect symmetry; increasing values indicate an increased asymmetry. Positive values indicate an asymmetry towards the right limb lameness and a negative value indicates an asymmetry towards the left limb lameness.

b A value of 0 indicates perfect symmetry, a value of 1 indicates maximum asymmetry towards the right limb and a value of −1 indicates maximum asymmetry towards the left limb.

### Data analysis

Open software (R‐Studio, version 3.3.1)^4^ was used for statistical analysis of the calculated symmetry parameters. For the sacrum, LTC and RTC clusters, a generalised linear model (function: glm) was used with the position of each cluster marker in the volume as an explanatory variable and the tested symmetry parameters (per stride) used as a response variable. To determine the position of each marker in relation to the treadmill coordinate system (*x*: left/right, *y*: cranial/caudal, *z*: vertical), the horizontal coordinates (i.e. *x*,* y*) were used to determine cranial/caudal and left/right misplacement. For LTC and RTC, the vertical plane (i.e. *y*,* z*) coordinates were used for determining cranial/caudal and proximal/distal misplacement. Since all horses had a baseline motion asymmetry measured at the reference marker, all the measured symmetry variables (response variable) were normalised to the baseline measured asymmetry, thus defining the reference symmetry for each parameter at the reference marker as zero. All models were also tested for a nonlinear relation between outcome and explanatory variables. The Akaike's information criterion was used to select the best model. Best fit of each model was evaluated by plotting the residuals vs. fitted values to ensure homoscedasticity. Normal distribution of the residuals was verified using Q‐Q plots. Model plots were generated using the package ggplot2. Linear regression lines were also generated (function: geom_smooth) for model outcome visualisation.

Since the hip‐hike difference needs both LTC and RTC markers for its calculation, the analysis was performed by testing misplacement on one side while on the opposite only the central reference marker was used.

## Results

Per horse, a total of 30, 28 and 27 valid strides were analysed, respectively. None of the models improved significantly by using polynomial (i.e. nonlinear) relations and therefore all results are presented using linear models.

### Sacrum

**Figure 2:**
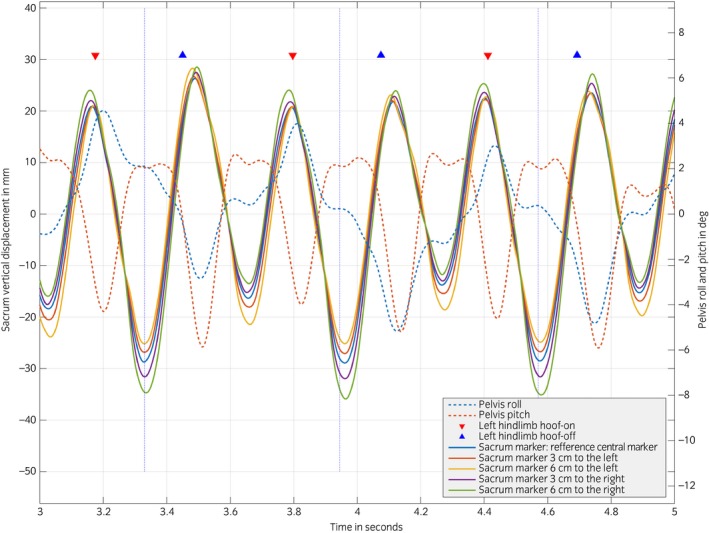
Tubera sacrale marker vertical displacement, pelvis roll and pitch angle related to left hindlimb midstance (vertical dotted blue line) for the Warmblood Horse. Midstance is defined as the moment of peak vertical ground reaction force. Pelvis roll (−): Left tubera coxae is below horizontal. Pelvis roll (+): Right tubera coxae is below horizontal. Pelvis Pitch (+): Lumbosacral extension. Pelvis Pitch (−): Lumbosacral flexion.

Figure 2 represents the timing of events between left/right displacement of the sacrum markers, limb‐midstance, pelvis roll and yaw, and hoof‐on/off events for one horse.

**Figure 3:**
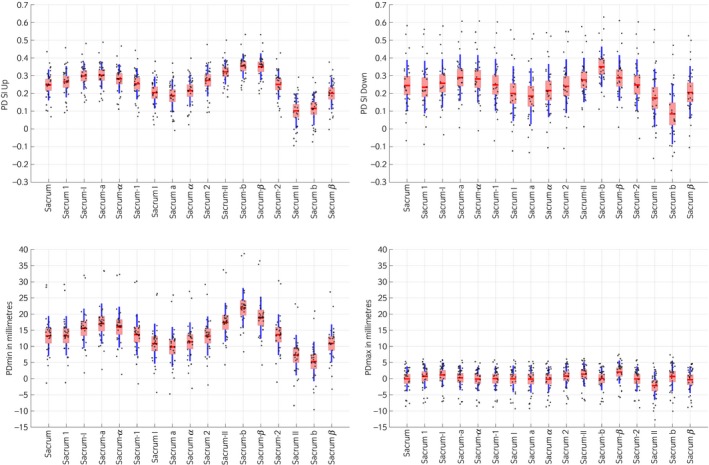
Plot of the symmetry parameters PDmin, PDmax, PD_SI__up_ and PD_SI__down_ for the Warmblood horse (30 strides). The red box indicates the standard deviation, the blue line the 95% SEM, the red line indicates the mean and all data points are scattered along each plot. Please refer to Table 2 and Fig 1 for marker names and corresponding orientation with respect to the central reference marker.

**Figure 4:**
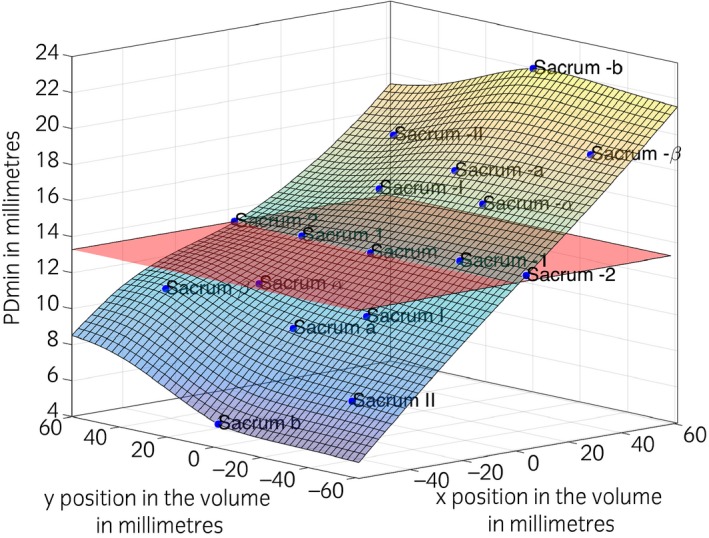
Change in PDmin (*z* axis) for the different marker placements for the tubera coxae. *x* and *y* axis represent the position of each marker in the global‐coordinate system (*y* positive to cranial and *x* positive to right). Each blue point represents one marker of the cluster as the average of all collected strides for the Warmblood horse (30 strides). The horizontal red plane represents the PDmin value of the central reference marker. The plane fitted along the different markers was generated using a thin‐plate smoothing spline method. Please refer to Table 2 and Fig 1 for marker names and corresponding orientation with respect to the central reference marker.

**Figure 5:**
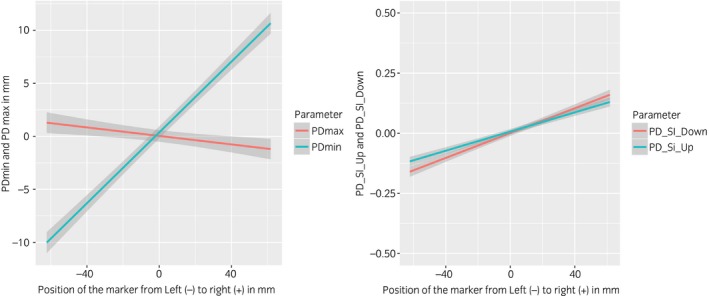
Left: Linear regression line with 95% confidence interval for PDmax and PDmin when markers are misplaced to the left or right. Right: Linear regression lines with 95% confidence interval for PD_SI__up_ and PD_SI__down_ when markers are misplaced to the left or right.

The baseline asymmetry calculated for each subject can be found in Table 1. Descriptive statistics of the symmetry parameters calculated for each marker are presented in Figures 3 and 4 for one horse (ID: 1). The model estimates including all horses indicated that for each cm the marker was misplaced to the left or right from the reference anatomical landmark, PDmin was affected by ±1.67 mm (95% CI 1.54–1.8 mm; P<0.001), PDmax by ±0.2 mm (95% CI 0.071–0.33 mm; P = 0.003) (Fig 4), PD_SI_up_ by ±0.019 (95% CI 0.017–0.022; P<0.001) and PD_SI_down_ by ±0.026 (95% CI 0.023–0.029; P<0.001) (Fig 5), depending on to which side the marker was misplaced (to the left or to the right).

For cranial/caudal misplacement, each cm away from the anatomical landmark affected PDmin by ±0.04 mm (95% CI −0.09 to 0.16 mm; P = 0.6), PDmax by ±0.008 mm (95% CI −0.13 to 0.12 mm; P = 0.9), PD_SI_up_ by ±0.0004 (95% C.I −0.002 to 0.003; P = 0.8) and PD_SI_down_ by ±0.001 (95% CI −0.002 to 0.004; P = 0.5), depending on the direction in which the marker was misplaced (cranially or caudally).

### Hip‐Hike

**Figure 6:**
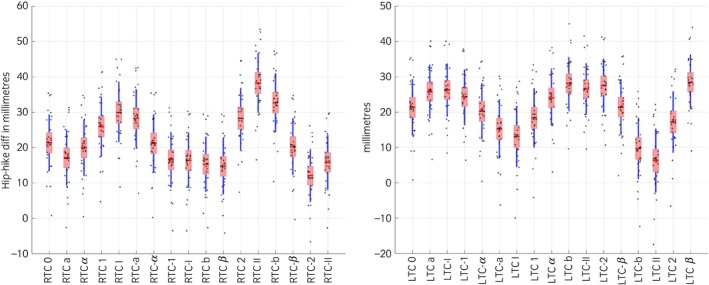
Plot of the symmetry parameter Hip‐Hike Difference when markers are misplaced on the LTC or RTC. The red box indicates the s.d., the blue line the 95% SEM, the red line indicates the mean and all data points are scattered along each plot. Please refer to Table 2 and Fig 1 for marker names and corresponding orientation with respect to the central reference of each marker.

For proximal‐distal misplacement, each cm away from the reference anatomial landmark (LTC/RTC) Hip‐Hike_Diff was affected by ±1.56 mm (95% CI 1.35–1.77 mm; P<0.001) (Fig 6). For cranial/caudal misplacement, each cm away from the reference anatomical landmark (LTC/RTC) Hip‐Hike_Diff was affected by ±0.82 mm (95% CI 0.66–0.97 mm; P<0.001).

## Discussion

The hypotheses that marker misplacement would affect symmetry parameters and this effect would be more pronounced when the misplacement is out of the sagittal plane are supported by the outcome of this study. We did not observe a considerable timing difference (on visual inspection of the signals) of peaks and valleys in the measured sacrum marker signals. We did observe a difference in amplitude of the signals and a difference in maximum and minimum vertical position when compared to the reference marker (Fig 5). This can be explained by the fact that the pelvis behaves like a rigid body 22; therefore, when using markers attached to the same rigid structure, vertical displacement events happen synchronously through the whole structure. For the calculated symmetry parameters of the sacrum, left/right misplacement had a considerably greater effect when compared to cranial/caudal misplacement. This is in line with previous research using sensors mounted along the midline of the horse, (between T6 and S3) 23, where small differences between locations of the sensor along the sagittal midline were reported for the calculated asymmetry parameters in most of the tested horses. In our study, the left/right misplacement PDmin was substantially more affected by misplacement than PDmax, as observed by the different model estimates ±1.67 mm for PDmin and ±0.2 mm for PDmax and as described for misplacement when using a pelvis mounted uniaxial accelerometer 14.

We hypothesise that this difference between PDmin and PDmax is mainly due to the soft tissue artefacts underneath the markers combined with the effect of pelvis roll. Functional surface EMG studies indicate that the *gluteus medius* muscle, located underneath the left/right misplaced markers, is active during the stride cycle, between the end of the swing phase until midstance during trot 24, 25. PDmin occurs during midstance. At this moment, the pelvis is most often slightly tilted (rolls) so that the tuber coxae of the stance limb is higher than that of the swinging limb (Fig 5). Hence, we argue that a sacral marker, misplaced to the right, would in right limb stance achieve its higher vertical position. This is possibly due to the combined effect of the roll of the pelvis and the upward push from the actively contracting *gluteus medius* of the stance limb.

PDmax is calculated at the pelvis’ maximum vertical position, which is the moment when the hoof is just about to strike the ground at the end of the swing phase. At this moment, the pelvis vertical position and roll is at its maximum with a clearly higher position of the tubera coxae of the protracting hindlimb. One might expect that this tilt would result in differences in vertical position between the several left/right misplaced markers, but in fact the difference between the markers is small (Fig 5). We hypothesise that during the maximum vertical position of the pelvis, even though electrical activity in the gluteal muscle is low, in the limb that has just pushed the body forward (i.e. the limb that is at the end of stance phase) 24, 25 there is a maximum extension of the hip joint 25 that passively compresses the gluteal muscle (and fat) on that side. This soft tissue movement will counteract the tilt created by the maximum pelvis roll and therefore result in only small vertical position differences across the markers observed (Fig 5).

Both PD_SI_up_ and PD_SI_down_ were affected by left/right marker misplacement, but to a different extent. Since these parameters are calculated based on the vertical range of motion difference between sides 21, both the upwards and the downwards pelvis movement ranges depend on the minimum position of the pelvis and, therefore, are affected by left/right marker misplacement.

The magnitude of the observed error remained small for small misplacements. Nevertheless, if the misplacement was big enough, the calculated symmetry parameters exceeded previously reported threshold values for PDmin (±3 mm 3) and hence led to a classification as lame. From our model estimates (Fig 4), a left/right misplacement of 3 cm resulted indeed in a false PDmin of ±5.01 mm, hence resulting in a type 1 error false positive result.

Misplacement of tuber coxae markers affects Hip‐Hike difference calculations and the effect is greater for the proximal‐distal misplacement. The observed artefact for proximal‐distal misplacement occurs on the tubera coxae peak just before hoof‐on on the misplaced side, which corresponds to the maximum pelvis roll towards that side (max tuber coxae elevation on the misplaced side) Also, during midstance on the misplaced side, the marker will achieve a lower position when compared to the reference marker, resulting in a systematic error of the calculated parameter. As there are yet no reference threshold levels for the pelvis Hip‐Hike difference, we cannot determine if the effect we measured might affect the clinical decision making. Nevertheless, attention should be given to properly place the markers in the correct anatomical location and in cases of horses with an asymmetrical pelvis conformation. In this situation, marker placement should be performed in a way that the distance to the tuber sacrale from each marker/sensor placed at the LTC and RTC is equal, avoiding any possible misplacement. This can be performed by measuring with a tape the distance between the sacrum marker/sensor and the LTC/RTC marker/senor.

In the present study, we used a small but diverse population of horses to avoid bias by any specific breed‐related morphological characteristics. It is unknown if different degrees of lameness that ultimately may alter the motion pattern of the pelvis 22, 26 could affect the model estimates we describe, but, as previously described, the horses included in this study were mildly asymmetrical (Table 1) and one of the horses even had a consistent pelvis movement asymmetry (PDmin = 13.3(±6.0) mm and Hip‐Hike Diff = 21.4(±8) mm). Further studies are needed to better understand the effect of marker misplacement on our investigated symmetry parameters in lame horses.

Although markers attached to the skin and moving relatively to the underlying bony structures do affect sensor orientation angle (Euler angles) 18, this effect was not yet tested for vertical displacement calculations. We believe that this orientation error effect would be more relevant for measurements using uniaxial accelerometer sensors 8, 14. Uniaxial accelerometers must be aligned with gravity to accurately measure displacement along the vertical axis. If incorrectly positioned, they will only measure a fraction of the total vertical acceleration, depending on their instantaneous orientation. This could in part explain the bigger effect of sensor misplacement described in another study using a uniaxial accelerometer 14. However, in that study the researchers used lame horses and sensors were misplaced between consecutive trials. Therefore, one cannot establish whether some of the observed differences may be related to between‐trial variation 8 or lameness‐related asymmetry.

More advanced sensor units such as an inertial measurements unit (IMU) sensor might be less prone to the orientation error, since their vertical orientation is corrected using three gyroscope sensors and three accelerometers 27. They therefore maintain the estimated vertical displacement in relation to the global‐coordinate system, resulting in the same vertical displacement as when measured using a 3D motion capture system. Therefore, the results presented here are also valid for sensors measuring vertical displacement in a global‐coordinate system 27, as long as the displacement estimated by the sensor is not under or over estimated. Thus, prior to the development of a sensor‐based system for objective lameness assessment, a validation study including agreement analysis with a 3D motion capture‐based system is imperative 27.

## Conclusion

Marker placement is important and due attention should be given to the instrumentation before performing objective gait assessment ensuring no markers/sensors are misplaced. For the sacrum and tubera coxae, left/right misplacement affected the measured symmetry parameters in a much greater magnitude in comparison to cranial/caudal misplacement. Overall, the observed error magnitude due to marker misplacement was small emphasising the repeatability of this technique. Nonetheless, if the left/right marker misplacement for the sacrum is for example greater than 3 cm, the observed error might exceed the previously described thresholds for lame horses and therefore result in a false positive or negative result. Since this study was carried out in a small population and using mildly asymmetrical horses, we cannot conclude that our results will hold true in clinically lame horses where a greater or smaller effect of marker misplacement might exist. As shown by our results, this could be due to changes in the pelvis rotation pattern. Nevertheless, the effect of lameness in pelvis rotation is known to be small 22. To study this, a kind of dose‐effect study would have to be conducted in a larger population of horses. However, this was beyond the scope of our study and here we aim to create awareness of the importance of correct marker placement.

## Authors’ declaration of interests

No competing interests have been declared.

## Ethical animal research

The study was performed with the approval of the Animal Health and Welfare Commission of the Canton of Zurich (permission No. 206/2010).

## Sources of funding

None.

## Authorship

F.M. Serra Bragança contributed to planning of the experiment, data processing, statistics and writing of manuscript. M. Weishaupt and T. Wiestner contributed to planning of the experiment, data collection and writing of manuscript. M. Rhodin, E. Hernlund, T. Pfau and R. van Weeren contributed to the writing of the manuscript and planning of the experiment.

## Manufacturers' addresses

1 Qualisys AB, Motion Capture Systems, Göteborg, Sweden.

2 Kagra, Switzerland.

3 MathWorks, 3 Apple Hill Drive, Natick, USA.

4 RStudio, Boston, Massachusetts, USA.
